# Isolation, Bioactive Potential, and Application of Essential Oils and Terpenoid-Rich Extracts as Effective Antioxidant and Antimicrobial Agents in Meat and Meat Products

**DOI:** 10.3390/molecules28052293

**Published:** 2023-03-01

**Authors:** Branislav Šojić, Sanja Milošević, Danica Savanović, Zoran Zeković, Vladimir Tomović, Branimir Pavlić

**Affiliations:** 1 Faculty of Technology Novi Sad, University of Novi Sad, 21000 Novi Sad, Serbia; 2 Faculty of Technology, University of Banja Luka, 78000 Banja Luka, Bosnia and Herzegovina

**Keywords:** terpenoids, natural antioxidants, antimicrobial agents, fresh meat, processed meat products

## Abstract

Using food additives (e.g., preservatives, antioxidants) is one of the main methods for preserving meat and meat product quality (edible, sensory, and technological) during processing and storage. Conversely, they show negative health implications, so meat technology scientists are focusing on finding alternatives for these compounds. Terpenoid-rich extracts, including essential oils (EOs), are remarkable since they are generally marked as GRAS (generally recognized as safe) and have a wide ranging acceptance from consumers. EOs obtained by conventional or non-conventional methods possess different preservative potentials. Hence, the first goal of this review is to summarize the technical-technology characteristics of different procedures for terpenoid-rich extract recovery and their effects on the environment in order to obtain safe, highly valuable extracts for further application in the meat industry. Isolation and purification of terpenoids, as the main constituents of EOs, are essential due to their wide range of bioactivity and potential for utilization as natural food additives. Therefore, the second goal of this review is to summarize the antioxidant and antimicrobial potential of EOs and terpenoid-rich extracts obtained from different plant materials in meat and various meat products. The results of these investigations suggest that terpenoid-rich extracts, including EOs obtained from several spices and medicinal herbs (black pepper, caraway, *Coreopsis tinctoria* Nutt., coriander, garlic, oregano, sage, sweet basil, thyme, and winter savory) can be successfully used as natural antioxidants and antimicrobials in order to prolong the shelf-life of meat and processed meat products. These results could be encouraged for higher exploitation of EOs and terpenoid-rich extracts in the meat industry.

## 1. Introduction

During the 21st century, meat production and processing have steadily enlarged worldwide [1]. According to FAO expectations, in 2030, meat production will range to nearly 373 million tons [2]. Generally, meat (pork, poultry, and beef) is marked as one of the main constituents of human diets owing to its high percentage of easily digestible proteins, vitamins, and vital minerals (iron, magnesium, phosphorus, potassium, and zinc) [1,3]. Fresh meat obtained after slaughtering animals (e.g., pigs, poultry, and cows) is treated at low temperatures under cooling or freezing conditions, and then packed in a vacuum or modified atmosphere without the addition of any synthetic preservatives [4]. On the contrary, meat products are obtained through a different method of processing (e.g., grinding, fermentation, smoking, drying, and heating) with the addition of various ingredients (e.g., salts, spices, emulsifiers, and preservatives) [4].

Fresh meat and processed meat products (raw, dry, and heat-treated meat products) contain a relatively high percentage of water, proteins, and lipids; therefore, they are easily prone to microbiological and chemical degradation, which leads to a loss of nutritive, sensory, and technological quality [5,6,7].

Oxidative reactions (lipid and protein oxidation) are the fundamental non-microbiological causes of quality deterioration in animal tissues, including meat and processed meat products [4,8,9], which leads to rancidity, discoloration, a decline in shelf-life and gathering of possibly toxic compounds that are risky to the health of customers [4,10]. Lipids, including triacylglycerides, phospholipids, and sterols, broadly range in both the intra- and extracellular space of meat. Phospholipids, as the main constituents of muscle cell membranes, comprise the highest percentage of unsaturated fatty acids; hence, they are the most susceptible to oxidation in comparison to other lipids [8,11]. Peroxides are the primary products formed during the free-step radical chain reactions of lipid oxidation. In the next phase, peroxides are subjected to the secondary processes of lipid oxidation, which leads to the formation of short-chain organic compounds such as aldehydes, ketones, and organic acids [9,12,13]. The intensity of lipid oxidation depends on countless causes, including pro-oxidant metal ions, temperature, air (oxygen), light, pH, degree of unsaturation, etc. [9]. Protein oxidation is one of the most advanced methods in meat quality assessment [4,13]. This process is contingent on the intensity of lipid oxidation. It is well known that protein and its derivates (free amino acids, peptides, and dipeptides) can react with free radicals formed during lipid oxidation [13,14]. Among the amino acids, the most predisposed to oxidative reactions are tyrosine, cysteine, histidine, phenylalanine, proline, methionine, lysine, arginine, and tryptophan. Protein oxidation causes the modification of the amino acid side chains generating the covalent intermolecular cross-linked protein and protein aggregation [14]. Moreover, protein modifications in muscle tissue, as an outcome of denaturation and proteolysis, induce loss of the sensory (color, flavor, and texture) and physico-chemical qualities of meat and processed meat products (reduction of proteolytic activity, loss of water-holding capacity, and decrease in biological functionality) [8,15,16].

Initial hygiene during the slaughtering process and the efficiency of applied processed techniques (e.g., salting, drying, and heating) are the leading causes of microbiological contamination in meat processing. Different types of microorganisms (bacteria, yeasts, and molds) can cause spoilage of meat and processed meat products and subsequently, various foodborne intoxications [17,18,19]. Spoilage bacteria (e.g., *Acinetobacter*, *Moraxella*, *Lactobacillus* spp., *Pseudomonas*, *Proteus* spp., and *Leuconostoc* spp.) are the most dominant in meat and processed meat products. These bacteria strains do not cause severe disease. However, in high concentrations, they affect degradation of the main constituents of meat (lipids and proteins) and finally affect the formation of disagreeable quality features (discoloration, slime and gas production, off-odors, and off-flavors) [18]. The degree of meat spoilage is the consequence of numerous factors such as initial hygiene, the temperature during different stages of processing, as well as the pH of meat and processed meat products [18]. Mataragas et al. [20] observed that pathogenic bacteria (e.g., *Salmonella* spp., *Escherichia coli* O157:H7, *Listeria monocytogenes*, *Campylobacter jejuni*, and *Clostridium* spp.) are principally responsible for foodborne diseases and food poisoning, but do not have a significant effect on sensory characteristics (e.g., color, flavor, and texture) of meat and processed meat products.

Recently, foodborne illnesses have been pointed out as crucial for controlling public health and economic development worldwide. Therefore, the microbiological deterioration of meat and meat products can be considered one of the main limitations in the modern meat industry [18]. Generally, delay of oxidative reactions and controlling bacterial growth are significant factors in quality preservation and improving the shelf-life of meat and processed meat products [10,13]. Using food additives is one of the critical methodologies for preserving the quality and prolonging the shelf-life of processed meat products [10]. The most abundant food additives in the modern meat industry are nitrites, which possess a strong antimicrobial and antioxidative potential. Conversely, these food additives from the subgroup of preservatives are marked as unhealthy [21].

Therefore, numerous investigations have been oriented toward finding natural alternatives for synthetic preservatives (e.g., nitrites) [21,22,23,24]. According to the high content of terpenoids (e.g., monoterpene hydrocarbons and phenolic terpenoids) with strong bioactive potential, essential oils (EOs) were be used as potential natural preservatives in different types of fresh meat and processed meat products. Furthermore, it is essential to apply adequate extraction procedures and optimize the process in order to obtain extracts or essential oils in high yield and quality in terms of terpenoid content. Therefore, the aim of this research is to provide a review of potent terpenoids present in medicinal and aromatic plants, insight about the extraction/distillation techniques which could be used for their isolation and summarize recent applications of these extracts/essential oils as natural antioxidants and/or antimicrobials in various meat products.

The search for scientific studies was carried in the Scopus database for original contributions that fitted in the scope of the present review. Essential oil* OR terpenoid extract* AND meat OR sausage were used as primary keywords in order to select articles published between “2008” and “2022”. This approach generated 427 results from which 90 were selected. Additionally, further research was carried out on the references of these selected papers, and 33 papers were selected for this review. Furthermore, EOs and extracts obtained by SFE which found applications as antioxidants and antimicrobials in meat and meat products were evaluated in terms of isolation by hydrodistillation techniques and supercritical fluid extraction.

## 2. Essential Oils and Terpenoid-Rich Extracts

EOs are a type of volatile fractions that possess unique and specific scents. These oils are extracted from aromatic plants using hydrodistillation or via cold pressing from citrus fruits peel. EOs are made of terpenoids, which are found in various parts of plants, such as glands, trichomes, and waxy channels. Furthermore, 85% of an EO is a mixture of different compounds such as terpenoids, esters, aldehydes, ketones, acids, and alcohols, while 15% of an EO is composed of minor compounds and trace elements [25]. For example, linalool is the predominant compound in the *Coriandrum sativum* EO comprising 84% of its content [26], while in *Salvia officinalis*, the major compounds are camphor (18%), α-thujone (17%), and eucalyptol (10%), and each minor compound is commonly present in less than 1% [27]. An EO’s chemical profile is inextricably linked to geographical origin, plant cultivar, development stage, climate, age, cultivation conditions, post-harvest processing of plant material, and the technique used for its isolation [28]. Paramount organic compounds found in EOs are terpenoids. Depending on the number of isoprene units, terpenoids are divided into several classes. These are monoterpenes, sesquiterpenes, and diterpenes. Considering that the number of isoprene units varies in terpenoids, those with more than 25 carbon atoms (sesquiterpenes, triterpenes, and tetraterpenes) are mostly found in fatty oils instead of EOs. Based on the fact that terpenoids represent one of the most complex compounds, rich in reactive functional groups, they are highly sensitive to oxidation. Therefore, hydrocarbon and oxygenated compounds are produced as main oxidation products, while nitrogen and sulfur are present in small percentages in EOs.

Due to the specific physico-chemical properties of terpenoids, the method used in their isolation, purification, and further application in food, cosmetics, and mostly in the pharmaceutical industry varies in terms of odor, polarity, volatility, and bioactivity. Antibacterial and antifungal activities are the reason why they have a wide range of uses; for example, they can be applied as direct natural antimicrobials for human and animal pathogens, and as alternatives for synthetic preservatives. Their specific physico-chemical properties are bioactivity, volatility, polarity, and odor; therefore, unique odor is the reason why terpenoids are used in the fragrance industry. For the same reason, terpenoids can have an impact on aroma in food, beverages, and drugs. EOs are highly potent antioxidant compounds which can be used as natural additives and at the same time have antimicrobial activity. Due to a multiplex chemical profile that makes EOs’ bioactivity impactful, they can manifest antiparasitic, allelopathic, anticarcinogenic, anti-obesity, immunomodulatory, anti-inflammatory, and antispasmodic effects [29].

Standardized EOs and their clear regulation are consequential, considering that there are many factors that can have an impact on the EOs’ chemical profiles. EOs and their components have GRAS status in the US, and they represent flavoring substances by Food and Drug Administration. In the EU, they are approved to be used in food packaging [30].

## 3. Isolation of Essential Oils

### 3.1. Hydrodistillation Techniques

Since EOs are used in different industrial sectors and separation techniques might significantly affect their chemical profiles, it is essential to find the best extraction procedure that could provide better results in terms of quality and adequate yields. Hydrodistillation (HD) is the commonly used method for the isolation of a pure EO consisting only of volatile compounds. This process is based on the contact of fresh or dry plant material with boiling water and/or steam, which is followed by the azeotropic distillation of water and the EO. Vapors are further cooled, and the EO and water are commonly collected as a two-phase liquid system that can be easily separated. This technique is used worldwide, and it is considered an economical method to produce EOs. HD is quite simple in its process, and this is one of the reasons why it is the most frequently used [31].

HD of terpenoids is affected by their volatility (boiling point) and solubility in water. When a raw material is immersed in boiling water, hydrophilic compounds are eluted from the surface of grinded plant cells and quickly distill with the water vapor. Part of an EO might be present inside the intact cells of the plant material, and these compounds firstly need to diffuse to the liquid phase and further distill in a subsequent step. Internal diffusion is faster for hydrophilic compounds, and the distillation of polar compounds would often occur first regardless of their boiling point. Distillation of an EO could be performed with water, steam, or a combined approach with steam and water, and the applied procedure, as well as process conditions, have a crucial effect on the yield and quality of an EO. Even though HD is considered to be most frequent in its usage and is a cost-effective technique, there are still weak points when it comes to the change in an EO’s chemical profile such as hydrolysis and thermal degradation which could occur during the process and diminish the quality of the EO. Additionally, HD is highly energy-consuming process because it involves heating and cooling the vapors. In addition, the process is time-consuming, and the contact between the plant and boiling water causes thermal degradation of EOs [31].

The aforementioned limitations of traditional HD were the main reasons for seeking innovative approaches in the development of new eco-friendly methods which could reduce solvents and the amount of time and energy used, as well as preserving the high quality of the product [32]. Recently, particular attention has been aimed towards utilization of microwave-assisted processes since they are able to address these challenges. Microwave heating, also known as dielectric heating, comes as a result of microwave irradiation (300 MHz–300 GHz), which is able to heat dielectric materials [31]. Molecular dipole rotation within the microwave field and ionic conduction are recognized as the main phenomena affecting the process. Polar molecules with high dipole moment tend to rotate with the effects of microwave field. This causes friction induced by colliding nearby dipoles, and the generated kinetic energy is used for heating which could further cause plant cell disruption and allow release of intracellular compounds in the liquid phase. Furthermore, ionic conduction caused by the electrophoretic movement of ions in the microwave field causes resistance of the medium, inducing the friction that further heats the solution. The strongest positive aspect of this technique is effective heat and transfer in the same direction caused by microwave irradiation, which is not the case with traditional HD. Absorption of microwaves and its effective conversion to thermal energy depends on the water content within the sample and dielectric constant of its compounds. Laboratory-scale microwave-assisted hydrodistillation (MWHD) could be performed at the modified set-up with a common microwave oven and glass apparatus (Unger- or Clevenger-type) for isolation of the EO. It is strongly suggested that MWHD could be used as an excellent alternative to traditional HD for EO isolation since this technique could provide significant improvements in terms of rapid energy transfer, efficient heating, fast recovery of the EO, and higher yield of the product.

A select recent comparison of HD and MWHD applications for the isolation of essential oils from medicinal and aromatic plants is provided in Table 1. The yield of the obtained EOs was provided only at optimized isolation conditions.

Chen et al. [33] performed comparative HD and MWHD of *Foeniculum vulgare.* Both techniques were observed, and the analysis of kinetic curves showed the gradual increase in EO yield. The techniques were performed in 140 min (HD) and 30 min (MWHD). After comparative analysis, the results supported MWHD because it is less time and energy consuming, and the higher separation efficiency and yield of the EO is noticeable.

Hassanein et al. [34] performed an in-depth comparison of HD and MWHD on selected medicinal plants from the Lamiaceae family. From the results, it can be concluded that MWHD provides a substantial reduction in distillation time, since it was three times faster than HD (HD: 180 min, MAHD: 60 min). Furthermore, MWHD was able to provide higher EO yield in all selected herbs, and these samples generally had a higher content of oxygenated terpenoids which are generally regarded as the more valuable EO compounds (Table 1).

Radivojac et al. [35] utilize *Salvia officinalis* herbal dust as the raw material for the recovery of the valuable EO. They compared HD and MWHD in terms of different levels of heating power in distillation kinetics, yield, chemical composition, antioxidant activity, and thermal behavior of the EO. Furthermore, they applied four models to describe the kinetics of the distillation process, and the highest yield (1.90%) was observed at 800 W. Results suggested that MWHD provides better results compared to conventional HD for producing EOs with a high content of oxygenated terpenoids and adequate bioactivity.

Another study from Radivojac et al. [36] considered HD and MWHD for the isolation of EO from peppermint leaves (*M. piperitae folium)*. In order to assess HD kinetics, the EO yield was obtained at different intervals within 120 min of process, applying two levels of irradiation power (205 and 410 W). On the other hand, MWHD was performed at the same time periods as HD with five levels of power (90, 180, 360, 600, and 800 W). The total HD yield was 0.73% (410 W), and MWHD showed a slightly higher EO yield (0.80%) with 800 W irradiation power. The EO yield can be improved more quickly using MWHD. However, applying higher microwave power can lead to changes in the chemical composition of an EO, because of the potential thermal destruction of compounds. The chemical characterization of terpenoids as a major class of lipophilic compounds in peppermint was obtained using GC–MS [37]. EOs obtained from HD and MWHD mostly contain monoterpenes such as menthol, menthone, isomenthol, isomenthone, and eucalyptol.

Mollaei et al. [38] suggested that optimized MWHD of *Ferulago angulata* provides the highest EO yield (6.50%) compared to EO obtained by HD (2.65%). This further indicated that MWHD could be the superior method for the isolation of this EO in terms of shortening process time, energy savings, reduced environmental hazard, and increased biological activities, such as antioxidant and cytotoxic activities, compared with the HD method [38].

Efficiency of MWHD was also observed in the isolation of *Myristicae arillus* EO, since the yield obtained by MWHD was 8.62%, achieved in 42 min, compared to 7.03% obtained by HD in 73 min [39]. The same study suggested that irradiation power is the most important MWHD parameter since EO yields obtained after 10 min at 300, 600, and 800 W were 2.68, 4.56, and 5.41%, respectively, and after 20 min, observed yields were 5.13, 7.39, and 6.83%, respectively.

**Table 1:** Select recent comparison of HD and MWHD applications for the isolation of essential oils from medicinal and aromatic plants.

Plant	Yield [%]		Major Compounds	Reference
	HD	MWHD		
*Foeniculum vulgare* Mill.	2.50	2.82	*trans*-Anethole	[33]
*Origanum majorana* L.	1.4	1.8	Terpinen-4-ol and *cis*-β-terpineol	[34]
*Mentha piperita* L.	2.2	2.6	Menthol and menthone	
*Mentha longifolia* L.	3.6	3.9	Isomenthone, pulgeone, and eucalyptol	
*Lavandula angustifolia* L.	1.2	1.4	Terpinen-4-ol, γ-terpinene, and *cis*-β-terpineol	
*Rosmarinus officinalis* L.	0.8	1	Camphor and eucalyptol	
*Thymus vulgaris* L.	1.8	2.1	Thymol and *o*-cymene	
*Salvia officinalis* L.	1.7	1.9	Viridiflorol, camphor, α-thujone, and eucalyptol	[35]
*Mentha piperita* L.	0.73	0.80	Menthol, menthone, isomenthol, isomenthone, and eucalyptol	[36,37]
*Ferulago angulata* Boiss.	2.65	6.50	Limonene and α-pinene	[38]
*Myristicae arillus*	7.03	8.62	1R-α-Pinene and β-pinene	[39]
*Organum vulgare* L.	5.81	7.10	Carvacrol	[40]

A few other comparative studies confirmed various advantages of MWHD over the traditional procedure [40]; however, it has been reported that the process had to be optimized in order to provide maximum efficiency. The recent commercialization of MWHD at an industrial scale and the aforementioned advantages in terms of EO yield, chemical profile, and bioactivity could lead to their utilization as food additives.

### 3.2. Supercritical Fluid Extraction

Organic solvent extraction by toluene, hexane, heptane, petroleum ether, methylene chloride, ethanol, methanol, etc., is occasionally used for the recovery of terpenoid compounds due to their lipophilic nature. This technique is commonly performed at lower temperatures compared to HD, which results in a reduced risk of chemical alterations caused by elevated temperature [31]. The majority of aforementioned organic solvents have a good affinity with terpenoids from EOs; however, they simultaneously cause the co-extraction of other lipid compounds which could result in poor selectivity and diminished quality of the extract. Further major issues with this approach are the considerably high cost, negative environmental impact, and potential human toxicity of the residues in the extracts.

Nowadays, supercritical fluid extraction (SFE) is used as an innovative technique for isolating EOs and lipophilic compounds from plants. It was developed to overcome the pitfalls of both solvent extraction and HD, and to reduce solvent consumption, save energy, decrease process times, improve selectivity and extraction yield, and prevent the target compound’s degradation triggered by high temperature. The main principle of SFE is application of fluids in their supercritical states which tremendously affect their physico-chemical properties. For example, high pressure cause increase supercritical fluids’ density, while their other properties such as viscosity, surface tension, and diffusivity are more similar to gases [41]. The characteristics of pressurized fluids increase in heat and mass transfer when viscosity is lower and diffusivity is higher. This leads to better fluid penetration into plant material pores and faster release of target compounds. With a relatively mild critical temperature (31.3 °C) and pressure (73.8 bar), carbon dioxide has been established as the most frequently used solvent in these processes. In addition to this, carbon dioxide has other properties desirable for the process. CO_2_ is non-flammable, non-explosive, available in a highly pure state, economical, non-toxic (GRAS), and inert [42].

The application of SFE requires utilization of specific high-pressure processing equipment including control of the main process parameters such as pressure, temperature, solvent flow rate, etc., since it could tremendously affect the efficiency and selectivity of the process. Solubility power of the solvent is influenced by pressure and temperature, and, as a result, they are regarded as the major SFE parameters. An increase in pressure is related to the increase in solvent density, which causes the improvement of solubility power. Consequently, the total extraction yield is higher, which is further related to the efficiency of the very process. Increased solubility power causes co-extraction of other lipophilic compounds, which results in reduced selectivity towards target compounds. In specific situations, coextracted compounds might have a beneficial impact on the bioactivity of obtained extracts [43]. In other situations, it might cause the dilution of active substances with concomitant compounds and cause complications in extract purification. On the other hand, the temperature increase has a negative impact on solvent density, and this often results in poor extraction yield. Solvent properties and the vapor pressure of the solute are strongly affected by temperature. Both solvent density and the vapor pressure of solute might improve bioactive compounds’ solubility, which contributes to an increase in total extraction yield [44]. It is very difficult to predict the amount of influence that temperature has on solvent density and vapor pressure of the solute; therefore, in order to determine the exact extraction yield, this process has to be experimentally monitored. This indicates that the appropriate choice of solvent selectivity can be performed by setting the pressure and temperature. According to the literature, a majority of SFE experiments are performed in 100–400 bar pressure and 40–60 °C temperature range [45], which allows 200 to 900 kg/m^3^ density of supercritical CO_2,_ making it an adequate solvent for lipid compounds. It is necessary to understand the thermodynamic and kinetic nature of the SFE process to ensure high extraction selectivity and a reduction of the possibility of non-target compounds’ coextraction [41]. For example, EO extracts rich in terpenoids might be commonly contaminated with non-volatile lipids, which might impact extract bioactivity and diminish quality. In addition to quality and extract bioactivity, the extraction spectrum of supercritical CO_2_ is problematic. SFE is limited to various lipid compounds with restricted applications on polar and moderately polar bioactives’ recovery. By adding different co-solvents, the problem of the selectivity and solubility power of supercritical CO_2_ can be solved. These co-solvents can be either polar or non-polar. Polar solvents are ethanol and methanol, whereas non-polar solvents are hexane and methylene chloride. The application of non-polar organic solvents has both positive and negative sides. For example, their low selectivity contributes to improvements in the total extraction yield. However, SFE could not be considered a green technique in this case. Ethanol is one of the most used co-solvents for SFE, considering its low miscibility in CO_2_, lower toxicity, and sample separation from the extract [41]. By using ethanol as an additional solvent, the spectrum of target compounds becomes wider, because SFE successfully isolates moderately polar bioactives. The important SFE parameters, apart from pressure and temperature, are solvent flow rate and extraction time. Solvent flow impacts mass transfer phenomena in the SFE process such as axial dispersion, convective mass transfer coefficient, and accumulation of extracted compounds in the supercritical phase [45]. Due to the constant input of fresh solvent, the increase in solvent flow rate improves mass transfer by increasing the gradient concentration. However, this factor could cause a decrease in total extraction yield in some cases. Mass transfer trigger by internal diffusion is prevented by exaggerated solvent flow, which also reduces solvent-matrix contact time. Having in mind that solvent flow can trigger unjustified high-solvent consumption, both solvent flow and its consumption have to be assessed for technological and economic aspects. Considering time consumption as a pivotal segment of the process, the extraction time commonly leads to an increase in total extraction yield; therefore, time can be inextricably linked to solvent consumption. Consequently, SFE optimization can be carried out by selecting the total extraction yield or target compound yield as responses [43]. SFE optimization is efficient for studies on a laboratory level and has a very limited application in industrial processes. Optimization is scarcely scalable on the industrial level since it is not economically achievable to carry out extractions because of the diffusion controlled period, and due to exaggerated time consumption. A recent study suggested a combined approach with the modeling of extraction kinetics in the first step and further optimization of the initial slope by either artificial neural networks (ANN) or response surface methodology (RSM) [46]. In addition to the aforementioned process parameters, the SFE of terpenoids relies heavily on plant matrix properties, particle size, distribution, and post-harvest material treatment. Previously mentioned factors have to be considered and assessed for each case.

Supercritical fluid separation of lipid extracts can be carried out by decreasing pressure and/or temperature in the separator which provides release of a gaseous fluid and allows recovery of solvent-free extracts. Instruments for SFE may contain more separators in order to fractionate the extract by adjusting various pressures and temperatures in those separators [47]. SFE with carbon dioxide at an industrial scale can be carried out with recirculation of the solvent, which can improve the efficiency of the process.

SFE requires complex equipment. This results in more investments, especially in industrial-scale plant processing. According to Prado et al. [48], the cost of a high-pressure processing plant with two extractors, each of 5, 50, and 500 L is approximately 100,000, 300,000 and 1,150,000 USD, respectively. Therefore, the price of the final product (SFE extract) could be a limiting factor for further utilization [49]. SFE has worldwide applications, especially in food production (decaffeination of coffee and tea), food ingredients (hops and aromas, colorants, vitamin-rich extracts, etc.), nutraceuticals, pharmaceuticals from natural products, and removal of pesticides from plant materials [49,50].

Supercritical carbon dioxide has been used for the isolation of different lipids from natural resources and has recently been established as a widely used method for EO isolation and extraction from medicinal plants [47]. Selected applications of SFE on the recovery of terpenoids from selected medicinal and aromatic plants are summarized in Table 2. A recent literature survey suggests that SFE certainly brings significant advantages compared to HD in terms of EO yield in particular (Table 2). The yield of extracts obtained using SFE was provided only at optimized isolation conditions.

The ANN approach was used for optimization of terpenoid recovery and antioxidant activity from *Salvia officinalis*, where pressure (100, 200, and 300 bar), temperature (40, 50, and 60 °C) and solvent flow rate (0.2, 0.3, and 0.4 kg/h) were investigated as independent variables [43]. Multi-response optimization predicted optimal conditions: 297.52 bar pressure, 44.39 °C temperature, and 0.4 kg/h solvent flow rate. The same study suggested that SFE provides a substantially higher yield (7.16%) compared to HD (1.80%).

SFE was compared with other conventional methods such as HD and Soxhlet extraction with organic solvents for the isolation of *Coriandrum sativum* EO, and SFE (100 bar and 40 °C) had an advantage over HD (0.60%) in terms of oil yield (1.52%), as well as in the yield of the predominant terpenoid, linalool (598.51 and 451.01 mg/100 g sample, respectively) [26]. The SFE of *C. sativum* was optimized by RSM in a further study investigating pressure (100–200 bar), temperature (40–70 °C), and CO_2_ flow rate (0.2–0.4 kg/h) as independent factors, and the predicted optimal conditions were 199.50 bar, 40.15 °C, and 0.396 kg CO_2_/h with the total extraction yield of 7.30% [51]. Various SFE studies suggested that extracts obtained at relatively mild conditions (100 bar and 40 °C) often have a high content of terpenoids, and this approach represents an excellent alternative to HD of pure EO [26]. According to Elgndi et al. [52], SFE at 100 bar and 40 °C provided a higher yield compared to conventional HD for both *Ocimum basilicum* and *Satureja montana*.

In addition to the aforementioned parameters, other SFE factors might significantly affect EO yield and should be considered in optimization studies.

For example, Shrigod et al. [53] used a central composite rotatable design to evaluate the effects of temperature (35–55 °C), pressure (100–300 bar), dynamic time (20–90 min), and particle size (0.2–1.0 mm) on the isolation of EO from *Mentha spicata*. EO content obtained using HD was very low (0.12–0.18%), while SFE provided significant improvements compared to traditional procedure since yield varied from 0.587 to 2.732%. Even so, the highest EO yield (2.732%) was obtained at the following set of SFE parameters: 45 °C, 200 bar, 55 min, and 0.2 mm; multi-response process optimization was done with the aim of simultaneously maximizing EO yield and carvone content in the extract, and the predicted parameters were 48 °C, 151 bar, 0.40 mm, and 37.5 min.

Ghasemi et al. [54] used 2^n−1^ fractional factorial design to optimize pressure, temperature, modifier volume, static, and dynamic extraction time as independent SFE factors used for *Myrtus communis* EO recovery. Results suggested that methanol could be efficiently used as a modifier of supercritical carbon dioxide since this factor had a significantly positive effect on extraction of the EO recovery; however, this approach demands further integration of another step of methanol removal in order to obtain solvent-free extract.

Radivojac et al. [36] compared standard Soxhlet, SFE, microwave-assisted, and ultrasound-assisted extraction in order to obtain the highest yield of lipid extracts. On 100 to 200 bars of pressure, a significant increase in extraction yield (2.62–3.52%) is noticed. It is concluded that a further increase in pressure did not change extraction yield (≈3.6%) significantly. Comparing extraction yield obtained by HD (0.73%) and SFE (2.62–3.52%), it can be concluded that SFE is more time consuming and enables a higher yield.

Ivanovic et al. [55] investigated the effects of different moisture content (10.5 and 28.4%) in raw material on kinetics and selectivity of SFE at 100 bar and 40 °C. Results showed an increase in monoterpene and sesquiterpene content and a decrease in content of waxy compounds in extracts isolated from wet *Helichrysum italicum* flowers (28.4% of water). Comparing these results with the chemical composition of extracts isolated from dried plant material (10.5% of water), it can be concluded that there are differences which could be explained by the modification of solvent power due to higher moisture content and an increased selectivity towards polar compounds.

Šojić et al. [57] researched the significance of adding by-products of *Thymus serpyllum* L. in ground pork patties. To recover lipid extracts rich in terpenoids, two SFE experiments were applied, SFE1 (100 bar at 40 °C) and SFE2 (350 bar at 50 °C). The total extraction yield obtained was 0.93% from SFE1 and 2.93% from SFE2, which is much more compared to the HD extraction yield obtained (0.15%). SFE products reduced lipid and protein oxidation and inhibited microbial growth in ground pork patties.

## 4. Bioactive Potential of EOs and Terpenoid-Rich Extracts

Plants such as rosemary, sage, thyme, oregano, basil, red pepper, lavender, peppermint, coriander, and clove have been used for a long time throughout history in food preparation and traditional medicine due to their specific flavors and biological activity. These plants are well known for their various effects—diuretic, antiseptic, analgesic, and antiallergenic. However, the most important effects that these plants have are antioxidant and antimicrobial effects [58]. In addition to phenolic compounds, which are well known for their antioxidant activity, a lot of attention has lately been given to EOs as contributors to preventing oxidative stress. Considering that oxidative activity cannot be prevented during the storage and processing of products in the food industry, adding antioxidants is required in order to prolong shelf life [59].

The antioxidative process starts with compounds that donate their hydrogen atom to trigger more stable radicals. After lipid oxidation as a three-phase process, free radicals which initiate a radical chain reaction are generated [60]. Antioxidants are necessary in order to prevent two free radicals from collision. This is achieved by donating a hydrogen atom. These compounds exert their activity through different mechanisms thanks to their ability to act as free radicals’ scavengers, electron donors, donors of H-atoms, regulators of enzyme activity, chelators of metal ions, and inhibitors of pro-oxidative enzymes [61]. Based on the mechanism used for preventing oxidation, there are two types of antioxidants. Preventive antioxidants include enzymes such as superoxide dismutase, glutathione peroxidase, glutathione reductase and catalase, which remove initially formed radical species. A second group of antioxidants are chain-breaking antioxidants which react with peroxyl radicals and in such a way prevent propagation in lipid oxidation [62].

Furthermore, when observing antioxidative effects at the cell level, organelles responsible for cell breathing, mitochondria, are the main sources of ROS in our organism. As a result of oxidative stress, the imbalance between generating ROS, and the antioxidative defense mechanism, various diseases are triggered in the human body [63]. Cells have their own mechanisms to avoid any kind of negative environmental stimuli. Regulating mitochondrial biogenesis is important because of the adenosine triphosphate (ATP) intracellular transfer of energy and cellular metabolism regulation [64]. Phenols are exogenous antioxidants that, due to their physicochemical characteristics, have a pivotal role in regulating intracellular pathways of mitochondrial biogenesis. Regulation involves removing damaged mitochondria and generating new mitochondria. The goal is to achieve mitochondrial homeostasis [64]. By activating peroxisome proliferator-activated receptor-*γ* coactivator- (PGC-), which oversees mitochondrial biogenesis, phenolic compounds have the ability to generate new mitochondria [65]. Due to the characteristics of phenolic compounds, it is possible to add antioxidant-rich extract to food products. Foods enriched with these antioxidative compounds have the ability to prevent diseases such as cancer and neurodegenerative and cardiovascular diseases [66]. For example, EOs from mint manifest antioxidative activity through phenolic acids, flavonoids, and unsaturated cyclic oxygenated terpenes [67]. Menthol and menthone, which are the main components of mint’s EOs, contain the hydroxyl radical (-OH) group. As a result, they conduct significant radical scavenging activity against hydroxyl radicals and hydrogen peroxide radicals. Based on the position of the alkyl group present in thymol and carvacrol, these compounds have the ability to neutralize free radicals [68]. Due to a similar phenolic structure, linalool, 1,8-cineole, geranial, neral, and citronellal contribute to the antioxidant properties of EOs.

It is well known that oxidative reactions (lipid and protein oxidation) in muscle tissues are the main limitation factors regarding the quality and shelf-life of meat and processed meat products. Namely, these oxidative reactions begin at the time of slaughter, when blood flow is interrupted and the metabolic processes are blocked [69]. Lipid oxidation is a complex process in which unsaturated fatty acids react with molecular oxygen via a free radical chain forming peroxides. The first auto-oxidation is followed by a series of secondary reactions, which lead to lipid degradation and the development of oxidative rancidity products [69]. Similarly to lipid oxidation, the protein oxidation process begins with the initiation stage of free radical formation and hydroperoxide generation before transitioning to the propagation stage of radical proliferation and transfer and concluding with the termination stage, which is summarized as the formation of non-reactive species. Free radicals are highly reactive and can directly react with protein molecules through hydrogen abstraction, coupling, oxygenation, and cleavage [14].

The intensity of lipid and protein oxidation in fresh meat depends on many other factors, such as the lipid and protein contents, fatty acid profile of muscle and fatty tissues, animal diet and lifestyle, and cooling conditions. The main factors affecting oxidative changes in processed meat products are the processing methods, storage conditions, types of ingredients, and presence and concentrations of pro- or antioxidants [69].

In addition to the antioxidant role, the components of EOs also exhibit strong antimicrobial activity [70]. Antimicrobial effects depend on the chemical composition of the oils and its main components. Bioactive compounds from EOs (thymol, carvacrol, linalool, and menthol) manifest their antimicrobial activities due to the presence of an aromatic structure with highly active functional groups [18].

The cell membrane of bacteria is the main barrier that antimicrobial agents must overcome in order to manifest their effects. Due to the fact that the structure of the cell membrane differs in Gram-positive and Gram-negative bacteria, the mechanisms of action of antimicrobial agents are different. The advantages of lipophilic components from EOs are that they pass through the lipid membrane of Gram-positive bacteria, which consists of a peptidoglycan layer [71]. After passing through the membrane, they can cause various effects such as cell wall destruction, membrane protein damage, increasing permeability, cytoplasm coagulation, reduction of proton motive force, and reduction of the intracellular ATP pool [72]. These mechanisms lead to lysis and cell death [73]. Gram-negative bacteria possess more complex outer membranes rich in lipopolysaccharide, which is the main reason why essential oils are more susceptible to Gram-positive bacteria [70].

The highest antimicrobial effects are found in phenolic compounds, among them monoterpenes (carvacrol, eugenol, and thymol) followed by aldehydes and ketones (β-myrcene, α-thujone, geranyl acetate), while the lowest effects are found in alcohols and hydrocarbons [74]. By decreasing pH value, carvacrol affects the loss of ATP [75]. Eugenol from *O. basilicum* can inhibit ergosterol biosynthesis, which is a specific component of the cell wall membrane. In this way, eugenol destroys the integrity of the cell membrane [74]. Eucalyptol which is present in rosemary, sage, and mint shows high antimicrobial activities against *Salmonella* spp., *Escherichia coli*, and *Listeria monocytogenes* [27]. Thymol has the ability to disintegrate the outer membrane of bacteria and in that way increase permeability of the cytoplasmic membrane to ATP. Sage EOs have antibacterial activity against Gram-positive and Gram-negative bacteria mainly due to the presence of camphor, β-thujone, α-thujone, eucalyptol, viridiflorol, and *trans*-cariophyllene [59]. Mint EOs with antimicrobial effects can also be used against microorganisms [67]. EOs isolated from mint contain menthol as the dominant compound, which increases permeability of bacteria cell membranes. Menthone, *cis*-caran, and eucalyptol have the same antimicrobial mechanism [76]. From the literature, mint EOs inhibited growth of Gram-positive and Gram-negative bacteria such as *Bacillus subtilis*, *Serratia marcescens*, *Pseudomonas aeruginosa*, and *Staphylococcus aureus* [77]. By using the whole oils instead of individual components, better antimicrobial activity can be achieved due to the synergistic action among them. For example, thymol in synergy with carvacrol, *p*-cymene, and γ-terpineol are able to modify the permeability of the bacterial cell membrane [78].

There are many positive sides when applying EOs in the food industry. EOs extracted from plants have proven to be safer, achieve better tolerance in the human body, and are cost-effective. Additionally, these EOs are lower in toxicity, have fewer side effects, and demonstrate better biodegradability and lower resistance [68].

## 5. Terpenoid-Rich Extracts as Natural Preservatives in Fresh Meat and Processed Meat Products

In this section, we summarize the most relevant studies carried out on the application of terpenoid-rich extracts obtained from the most frequently used spices and aromatic herbs in meat processing: black pepper, caraway, *Coreopsis tinctoria* Nutt., coriander, garlic, oregano, sage, sweet basil, thyme, and winter savory (Table 3).

Black pepper (*Piper nigrum* L.) is one of the most dominant flavoring agents in meat processing [79]. With a relatively high percentage of terpenoids (limonene, α- and β-pinene, and caryophyllene), EOs isolated from black pepper (BPEO) display a strong antioxidative effect, as well as a preservative effect against a broad spectrum of microorganisms [79]. BPEO was added as a natural preservative in fresh pork loin at concentrations from 0 to 0.5%. All batches were stored at 4 °C for 9 days [79].

This study showed that BPEO delayed lipid oxidation and reduced the growth of *Enterobacteriaceae* and *Pseudomonas* spp. in fresh pork meat. In the study by Bi et al. [93], the influence of coating with BPEO (0.05 and 0.1%) on the lipid oxidation and sensory quality (aroma) of *Jinhua* ham (the dry-cured meat product) was examined. The authors suggested that using BPEO has a strong potential to suppress lipid oxidation and enhance the sensory acceptability of *Jinhua* ham during long-term storage (4 months at room temperature). Generally, the results suggest that BPEO could be used as a natural antioxidant in dry-cured meat products.

The essential oil isolated from caraway (*Carum carvi* L.)—CEO is widely used as a bioactive component in pharmaceutical processing [94]. In vivo studies confirmed the preservative potential of CEO, and it was recommended as a potential preservative and quality enhancer in different foodstuffs. The strong biopotential of CEO can be related to a high percentage of terpene, limonene, and ketone carvone [94]. A strong antimicrobial potential against various fungal and bacterial species were determined by Hromiš et al. [95] and Kocić-Tanackov et al. [96]. Krkić et al. [94] investigated the influence of the coating chitosan-biopolymer film enriched with CEO on the quality and shelf-life of traditional fermented sausage produced in Serbia (Petrovská klobása). This product was stored. The results of the study mentioned above suggested that a chitosan-caraway coating can contribute to delaying lipid oxidation and to preserving the required sensory attributes (odor and taste) of Petrovská klobása during a long storage period (for 5 months at 10 °C). Šojić et al. [80] investigated the antioxidant effect of CEO (1, 2 and 5 µL/g) in cooked pork sausages. The addition of CEO (all three concentrations) significantly reduced TBARS values compared to the control. Generally, the obtained results show that CEO can be used as a natural preservative agent in dry and heat-treated meat products.

EO isolated from *Coreopsis tinctoria* Nutt. (CTNEO) possesses a strong preservative potential in the food industry according to its high content of phenolic acids and flavonoids [81]. CTNEO was used in order to improve the quality and shelf-life of fermented sausages produced with the usage of horse meat. Namely, the group of scientists [81] applied CTNEO and its microcapsules (CTNEOM) in the basic formulation of this meat product. Both preservative types were added at 0.312%, corresponding to CTNEO’s minimally inhibitory concentration (MIC) towards *Morganella morganii* (the leading microorganism responsible for biogenic amines-formation) and *Enterococcus faecalis*. This study showed that CTNEO, mainly CTNEOM, could effectively prevent lipid oxidative changes, *Enterobacteriaceae* growth, and biogenic amines (histamine, tyrosine, cadaverine, putrescine, and tryptamine) formation in fermented sausages of horse meat [81]. Also, CTNEOM provided better sensory quality (color and flavor) in the final products. Generally, encapsulation of EOs allowed the usage of relatively high content of natural preservatives without negatively impacting the sensory quality of final products.

Coriander (*Coriandrum sativum* L.) is an ancient aromatic herb with significant nutritional and medicinal properties. Because of the synergistic effect among terpenoid compounds (linalool, limonene, camphor, and geraniol), essential oil from coriander (COEO) is categorized as a strong preservative and flavoring agent in food and biotechnology [26]. In our previous study [82], we investigated the influence of COEO (0.075–0.150 µL/g) addition on the quality and shelf-life of cooked pork sausages, which were produced with different levels of sodium nitrite (0, 50 and 100 mg/kg) and subjected to cold storage for 60 days. Also, in this study, we evaluated the possibility of using COEO as a partial substitute for sodium nitrite. The results of this study presented that COEO could be used as a partial replacement for sodium nitrite (50 mg/kg) at a concentration of 0.12 μL/g. Generally, the synthetic additive (sodium nitrite) and COEO (0.12 μL/g) enabled optimum quality for the final product during 52 days of storage. The group of scientists [83] observed the influence of COEO addition (0.02%) on the microbiological profile of fresh minced beef stored at 0.5 ± 0.5 °C and 6 ± 1 °C for 15 days. González-Alonso et al. [83] detected that COEO addition decreased the growth of *Enterobacteriaceae* (by up to approx. 1–2 log cycles related to the controls) in fresh meat products.

Garlic (*Allium sativum*), an ancient spice and aromatic plant from Alliaceae family, is widespread worldwide. Fresh bulbs and the essential oil of garlic (GEO) possess strong antimicrobial attributes; hence, they are widespread in foodstuffs, especially in meat processing [97]. The most dominant compounds of GEO are sulfuric-terpenoids, particularly allicin, which is responsible for most of the antimicrobial and antioxidant activity and flavor. The preservative effects of different antimicrobials (GEO, allyl isothiocyanate, and nisin) on the shelf-life of fresh sausages previously inoculated with *E. coli* O157:H7 were assessed by Araújo et al. [84]. The authors suggested that the combinations of these antimicrobials effectively reduced the growth of *E. coli* and lactic acid bacteria, and maintained the red color of fresh sausages during storage (for 20 days at 6 °C). Najjaa et al. [98] examined the influence of microencapsulated GEO in preserving minced beef meat. The results exhibited that microencapsulated GEO at a concentration of 20% efficiently reduced the following foodborne pathogenic bacteria: *Escherichia coli*, *Salmonella* spp., and the sulfite-reducing anaerobes. In another study, Esmaeili et al. [97] examined the effect of chitosan and whey protein film impregnated with GEO on the shelf-life of cooked beef sausages kept in refrigeration conditions for 50 days. This study presented that the GEO application in active packaging reduces microbiological growth and lipid oxidation in cooked sausages.

Sage (*Salvia officinalis* L.) and its EO (SEO) have a long tradition in medicine and food preparation as flavoring agents. SEO contains broad spectrum of bioactive compounds, including monoterpene ketones (e.g., α-thujone, β-thujone, and camphor) and diterpene polyphenols (e.g., epirosmanol, carnosol, and carnosic acid) with significant preservative potential [27,43]. SEO and sage extracts (water and ethanol) were used as potential natural preservatives in low-pressure mechanically separated meat (MSM) processing during frozen storage (for 9 months at −18 °C) [85]. The quality and shelf-life of MSM samples were examined based on microbiological analyses and TBARS test (thiobarbituric acid reactive substances). This study exhibited that SEO efficiency reduced lipid oxidation of MSM. In the case of microbiological stability, it was reported that all three types of extracts reduced the following bacteria strains: *Enterobacteriaceae*, coliforms, mesophilic, and psychrotrophic bacteria. It should be highlighted that the most powerful inhibitory effect for all tested bacteria was demonstrated by SEO, as a consequence of its relatively high content of terpenoid compounds (monoterpenes and diterpenes).

In the case of meat products, Šojić et al. [27] assessed the effect of SEO and sage extract (SE) obtained using SFE with carbon-dioxide at concentrations of 0.050, 0.075, and 0.100 µL/g on the quality (chemical, microbiological, and sensory) of fresh pork sausages. Extracts were isolated from by-products of the filter-tea industry in Serbia. This study presented that SEO and SE reduced the products of lipid oxidation and microbiological growth in fresh pork sausages during cold storage (for 8 days at 3 ± 1 °C). Generally, compared with conventional SEO, SE allowed better antimicrobial potential and sensory acceptability in fresh pork sausages, proposing the benefit of the novel SFE technique. Additionally, SEO usage at deficient concentrations (0.4 and 0.6 μL/g) and SE (0.4, 0.6 and 1 μL/g) can prevent the spread of *E. coli* and extend the shelf-life of ground pork meat up to 8 days at cold storage (4 °C) [86]. Hence, SEO, and particularly SE, can be used as effective natural preservatives in fresh sausages.

Oregano (*Origanum vulgare* L.) and its EO (OEO) have been revealed to contain terpenoid compounds (carvacrol and thymol) with strong preservative effects against microbiological growth and oxidative reactions in meat and meat products [87]. OEO has been marked as one of the most potent EOs. Hence, Shange et al. [87] applied OEO at 1% (*v/v*) in black wildebeest meat (*biceps femoris*) in order to determine its preservative effect during refrigeration storage. This EO powerfully reduced lipid oxidation (expressed by malondialdehyde) and microbiological growth (total viable counts, lactic acid bacteria, and coliform counts) in black wildebeest meat. Also, Schirmer and Langsrud [99] determined that OEO inhibited *Salmonella* in fresh pork meat.

Sweet basil (*Ocimum basilicum* L.) is one of the most famous medicinal and aromatic plants from the Lamiaceae family. Basil essential oil (BEO) can be used as a natural additive in meat and processed meat products [88,96,100], because it contains high-potency terpenoids, included linalool and eugenol. In the research of Gaio et al. [88], the antibacterial activity of BEO (0.25–1.00 mg/g) in fermented sausages was examined. Gaio et al. [88] concluded that BEO efficiency reduces the growth of foodborne pathogenic bacteria, *Staphylococcus aureus*, during 30 days of storage at 22 °C.

Stojanović-Radić et al. [100] examined the influence of the addition of BEO on *Salmonella* Enteritidis growth and physico-chemical properties of fresh chicken meat during cold storage (for 3 days at 4 and 18 °C). Application of BEO reduced the population of *S.* Enteritidis, and decreased lipid oxidation (TBARS test), without a negative effect on the odor and flavor of the treated chicken meat. Recently, Kocić-Tanackov et al. [96] evaluated the antifungal effect of BEO and CEO against *Penicillium carneum* FEMK2 and *P. polonicum* FEMK5, isolated from Serbian fermented sausages, in vitro and in vivo. The authors suggested that CEO presented a better antifungal effect in vitro than BEO. Also, the application of EOs on the surface of Serbian fermented sausages artificially inoculated with conidia of *P. carneum* and *P. polonicum* reduced molds during the storage period. Thus, the obtained results suggest that BEO and CEO can be used as effective antifungal substances to protect fermented sausages.

Thyme (*Thymus vulgaris* L.) and its EO (TEO) comprise many bioactive compounds, including carvacrol and thymol, with a strong preservative effect. The effect of TEO addition on microbiological growth and biogenic amines formation in fermented sausages of horse meat was evaluated by Huang et al. [89]. The authors determined that TEO efficiently reduced *Enterobacteriaceae* counts and biogenic amines formation in this type of meat product.

Winter savory (*Satureja montana* L.) and its EO (WSEO) comprise a strong antioxidative and antimicrobial agent from the group of phenolic terpenoids, e.g., thymol and carvacrol. Hence, they are widespread in the food and pharmaceutical industries [91]. De Oliveira et al. [90] tested the influence of WSEO supplementation (7.80, 15.60 and 31.25 μL/g) on the color, microbiological growth, and oxidative status (TBARS test) of mortadella cooked pork sausages during storage (for 30 days at 25 °C). Mortadella was manufactured with three sodium nitrite levels (0, 100, and 200 mg/kg). De Oliveira et al. [78] observed that the sausage manufactured with 100 mg/kg of nitrite and without WSEO had a similar TBARS value to the sausages manufactured without sodium nitrite and with 15.60 and 31.25 μL/g of WSEO. This result suggested that WSEO could be a valuable alternative to sodium nitrite in heat-treated meat products. Also, de Oliveira et al. [90] showed that WSEO, in combination with sodium nitrite (100 mg/kg), declined the growth of *Clostridium perfringens* type A. Moreover, Šojić et al. [91] investigated the influence of WSEO and winter savory extracts obtained by SFE (0.075 and 0.150 µL/g) on the shelf-life of fresh pork sausages stored at 3 ± 1 °C for 8 days. Both extracts reduced the formation of malondyaldehide (TBARS test) and microbiological growth in the final product. It should be underlined that the extract obtained using SFE had a more potent antioxidant and antimicrobial potential than WSEO, undoubtedly due to a higher level of coextracted non-volatile lipids. It has been recognized that terpenoid-rich extracts of winter savory could be used as natural preservatives in processed meat products. Similarly, Jokanović et al. [92] determined a strong protective (lipid and protein oxidation) effect of WSEO in precooked pork chops. WSEO were obtained using hydrodistillation and SFE. The higher antioxidative potential was determined in the sample produced with WSEO obtained using SFE. Hence, the optimization of process extraction is essential to maximize the antioxidant activity.

In order to suppress oxidative reactions in meat and processed meat products, EOs and terpenoid-rich extracts could be used as natural antioxidants. Generally, the antioxidant and antimicrobial potential of EOs and terpenoid-rich extracts in meat and processed meat products depend on their concentrations, chemical profile, as well as quality characteristics of meat (e.g., pH, temperature, moisture, fat, and protein contents) and procedures during the meat processing (e.g., grinding, mixing, fermentation, drying, and heating) [70]. EOs, tocopherols, flavonoids, and phenolic acids have vigorous H•-donating activity or high radicals-absorbance capacity [69]. Some phenolics prevent free radical generation and the formation of reactive oxygen species, while others scavenge free radicals and chelate pro-oxidants (transition metal) in meat and processed meat products [101]. The antioxidant potential of these natural compounds (phenolics) depends on their structure and the distribution of functional groups in these structures. Also, phenolic diterpenes, one of the main bioactive compounds in EOs, act as iron chelators and eliminate peroxyl radicals, especially in meat products with a higher fat content [69]. The most dominant terpenoids of EOs and terpenoid-rich extracts are carvacrol, thymol, linalool, limonene, camphor, ɑ-thujone, etc. [70]. Carvacrol and thymol were registered as the most dominant terpenoid compounds of oregano, thyme, and winter savory extracts, while linalool was the most dominant terpenoid of coriander and basil extracts. These terpenoids efficiently reduce lipid oxidation in meat products by scavenging free radicals and inhibiting lipid peroxidation ability [78].

In addition, carvacrol and thymol are marked as terpenoid phenols with significant antimicrobial potential. The high content of carvacrol and thymol causes the increased permeability of cell membranes. It simultaneously causes a reduction in pH gradient across the cytoplasm membrane, as well as the inhibition of ATP synthesis, and finally, the death of bacterial cells [3]. Concerning linalool, since it is the major component of coriander and basil extracts, its bactericidal action could be explained by the fact that this compound may act on the bacteria’s cell wall [102]. The strong protective effect of black pepper and caraway extracts could be related to the presence of limonene. Namely, Gupta et al. [103] observed that exponentially growing *E. coli* cells were inactivated by limonene at the concentration of 2000 μL/L. The mechanism of bacterial inactivation was observed to be the Fenton-mediated hydroxyl radical formation which leads to the oxidative DNA damage of the bacterial cell.

Moreover, interactions between the different terpenoids enable the preservative effect of EOs and terpenoid-rich extracts. The chemical composition of meat and meat products also affects the preservative effect of EOs and terpenoid-rich extracts. High contents of lipids and proteins in meat or meat products protect the bacteria strains from the action of the EO in some way. The susceptibility of bacteria to the preservative effect of EOs and terpenoid-rich extracts also appears to increase with a decrease in the pH of the food, the storage temperature, and the amount of oxygen within the packaging [70].

Although EOs and terpenoid-rich extracts possess robust preservative effects, there are a few limitations regarding the application of these natural extracts in meat and meat products. Firstly, it is well known that some medicinal and aromatic plants at high concentrations could be toxic to humans. For example, thujones above 0.5 mg/kg affect acute toxicity. Hence, Šojić et al. [27,91] limited the usage of sage EOs and sage SFE extracts to below 0.15 µL/g in fresh sausages. Likewise, the high concentrations of natural extracts, mainly terpenoid-rich extracts, provide undesirable sensory characteristics in the final products. Hence, it is crucial to find a compromise between effective doses of natural extracts, including EOs and terpenoid-rich extracts, and the safety and sensory acceptability of the flavored food. A study by Kahraman et al. [104] reported that concentrations of rosemary EO higher than 0.2% were not acceptable because of the sensory properties they imparted to the poultry meat. Moreover, Šojić et al. [27] determined a more robust limit for sensory acceptability of EOs extracts isolated from sage and winter savory (≤0.150 µL/g).

Finally, the economic implication of using EOs and terpenoid-rich extracts should also be considered [48]. Namely, due to the high cost of investment and labor, terpenoid-rich extracts obtained using SFE are limited for application in the food sector. However, novel investigations suggested that this technique provides a higher yield of terpenoid-rich extracts with strong preservative effects in a relatively small concentration in meat processing [27,91]. Also, several studies confirmed that terpenoid-rich extracts obtained using SFE possess a stronger preservative effect and better flavoring effects than EOs obtained using conventional HD technique [27,57,91,92].

## 6. Conclusions

In the past decade, there has been an enormous demand for natural substances primarily because of adverse toxicological reports on many synthetic compounds. Hence, many food and pharmaceutical engineering scientists have oriented themselves toward the isolation and purification of emerging plant extracts and, finally, their further application in foodstuffs. EOs can be extracted using conventional (e.g., HD and Soxhlet extraction) or novel extraction techniques (e.g., microwave-assisted and supercritical fluid extraction). SFE showed tremendous advantages over hydrodistillation techniques, which could be observed in the yield, quality, and bioactive potential of obtained extracts and terpenoid-rich fractions. Also, the results of these investigations suggested that terpenoid-rich extracts, including EOs obtained from several spices and medicinal herbs (black pepper, caraway, *Coreopsis tinctoria* Nutt., coriander, garlic, oregano, sage, sweet basil, thyme, and winter savory) can be successfully used as natural antioxidants and antimicrobials in order to prolong the shelf-life of meat and processed meat products. Also, terpenoid-rich extracts can be used as partial substitutes for nitrites and packaging ingredients to improve the quality and shelf-life of meat and processed meat products. Although EOs and terpenoid-rich extracts possess a robust preservative effect, there are a few limitations regarding the application of these natural extracts in meat and meat products. High concentrations of extracts from different plant materials provide undesirable sensory characteristics in the final products and possess a toxicity potential for humans. Hence, optimizing EOs and terpenoid-rich extracts in meat processing is essential. Further investigations need to be conducted to develop the encapsulation of isolated terpenoid extracts from different plant materials, with the aim of more straightforward application in the meat industry.

## Figures and Tables

**Table 2:** Recent applications of SFE on the recovery of terpenoids from selected medicinal and aromatic plants.

Plant	Investigated SFE Parameters		Yield [%]		Reference
			SFE	HD	
*Salvia officinalis* L.	100–300 bar 40–60 °C 0.2–0.4 kg CO_2_/h		0.59%–7.16%	1.80%	[43]
*Coriandrum sativum* L.	100 and 300 bar 40 °C 0.2 CO_2_/h		1.52% and 8.88%	0.60%	[26]
*Coriandrum sativum* L.	100–200 bar 40–70 °C 0.2–0.4 kg CO_2_/h		0.59%–7.00%	-	[51]
*Ocimum basilicum* L.	100 and 300 bar 40 °C 0.2 kg CO_2_/h		1.56% and 2.07%	0.67%	[52]
*Satureja montana* L.	100 and 300 bar 40 °C 0.2 kg CO_2_/h		1.50% and 4.02%	1.15%	[52]
*Mentha spicata* L.	100–300 bar 35–55 °C 20–90 min 0.2–1 mm		0.587%–2.732%	0.12%–0.18%	[53]
*Myrtus communis* L.	100–350 bar 10–40 min 0–150 μL modifier		0.5%–6.3%	0.47%	[54]
*Mentha piperita* L.	100–400 bar		2.62%–3.52%.	0.73%	[36]
*Helichrysum italicum* (Roth) G. Don fil.	100 bar 40 °C 10.5 and 28.4% moisture		2.45 and 2.78%	0.23%	[55]
*Ocimum basilicum* L.	100–300 bar 60 °C 0.2 CO_2_/h 4 h		0.657%–2.206%	0.565%	[56]
*Thymus serpyllum* L.		100–350 bar 40–50 °C 0.3 kg CO_2_/h	0.93%–2.93%,	0.15%	[57]

**Table 3:** Recent applications of SFE on the recovery of terpenoids from selected medicinal and aromatic plants.

Plant	Extract	Dose	Fresh Meat/Processed Meat Products	Storage	Effect	Reference
*Piper nigrum* L.	EO	0.1, 0.5%	Fresh loin	4 °C, 9 days	RMG	[79]
*Carum carvi* L.	EO	1, 2, 5 µL/g	Cooked sausages	4 °C, 1 day	DLO	[80]
*Coreopsis tinctoria* Nutt.	EO	0.312%	Horse meat fermented sausage	10–12 °C, 28 days	DLO, RMG	[81]
*Coriandrum sativum* L.	EO	0.075–0.150 µL/g	Cooked sausage	4 °C, 1 day	DLO, RMG	[82]
	EO	0.02%	Fresh ground chicken meat	6 °C, 15 days	RMG	[83]
*Allium sativum* L.	EO	125 µL/kg	Fresh sausage	6 °C, 20 days	RMG	[84]
*Salvia officinalis* L.	EO	2%	MSM	−18 °C, 9 months	RMG	[85]
	EO, SFE	0.05–0.1 µL/g	Fresh sausage	4 °C, 8 days	DLO, RMG	[27]
	EO, SFE	0.2–0.6 μL/mL	Minced pork meat	4 °C, 3 days	RMG	[86]
*Origanum vulgare* L.	EO	1% (*v*/*v*)	Frozen black wildebeest meat	2 °C, 9 days	RMG	[87]
*Ocimum basilicum* L.	EO	0.25–0.1 mg/g	Fermented sausage	22 °C, 30 days	RMG	[88]
*Thymus vulgaris* L.	EO	-	Horse meat fermented sausage	10–12 °C, 28 days	RMG	[89]
*Satureja montana* L.	EO	0.78–3.125%	Cooked sausage	25 °C, 30 days	RMG	[90]
	EO, SFE	0.075–0.150 µL/g	Fresh sausage	4 °C, 8 days	DLO, RMG	[91]
	EO, SFE	0.200 µL/g	Precooked pork chops	4 °C, 6 days	DLO	[92]

EO—essential oil; SFE—supercritical fluid extract; DLO—decrease in lipid oxidation; RMG—reduction of microbiological growth.

## Data Availability

Not applicable.
